# Relationship Between Social Deprivation and Access to Catheter Ablation for Atrial Fibrillation: A Population-Level Study

**DOI:** 10.1016/j.jacadv.2024.101400

**Published:** 2024-11-18

**Authors:** Andriy Katyukha, Feng Qiu, Denis Qeska, Ragavie Manoragavan, Harindra C. Wijeysundera, Christopher C. Cheung

**Affiliations:** a Temerty Faculty of Medicine, University of Toronto, Toronto, Ontario, Canada; b University of Toronto Department of Medicine, Toronto, Ontario, Canada; c Schulich Heart Program, Sunnybrook Health Sciences Centre, University of Toronto, Toronto, Ontario, Canada; d ICES, Toronto, Ontario, Canada

**Keywords:** arrhythmia, equity, marginalization, procedure

## Abstract

**Background:**

Access to catheter ablation for atrial fibrillation (AF) may vary due to social deprivation.

**Objectives:**

This study sought to characterize the correlation between our outcomes of interest (rates of AF diagnoses, ablation referrals, and procedures) and the association between social deprivation and our outcomes.

**Methods:**

Rates and correlations of AF diagnoses, ablation referrals, and procedures were reported across 49 census divisions in Ontario, Canada. We used the Ontario Marginalization Index to determine the relationship between dependency, material deprivation, ethnic concentration, and residential instability and our outcomes.

**Results:**

Between April 2016 and March 2020, there were 146,366 patients diagnosed with AF; 6,506 patients were referred for ablation; and 4,673 patients underwent de novo ablation. The median age was 72 years (IQR: 61-81 years; 45% female) for the AF cohort and 62 years (IQR: 55-69 years, 33% to 34% female) for the referral and procedure cohorts. There was geographic variation and a weak concordance between AF diagnoses, ablation referrals, and procedures (correlation coefficients 0.33-0.36). Increased material deprivation was associated with more AF diagnoses (rate ratio [RR]: 1.13), but fewer ablation referrals (RR: 0.49) and procedures (RR: 0.48). Increased residential instability was associated with more AF diagnoses (RR: 1.02), but fewer ablation referrals (RR: 0.63) and procedures (RR: 0.64). Higher ethnic concentration was associated with fewer AF diagnoses, ablation referrals, and procedures.

**Conclusions:**

In a jurisdiction with universal health care, greater material deprivation and residential instability were associated with more AF diagnoses but less access to ablation, suggesting substantial social gradients in equitable access to AF care.

Atrial fibrillation (AF) is the most common sustained cardiac arrhythmia and is associated with an increased risk of stroke, heart failure, and hospitalization.^1^, ^2^, ^3^ Catheter ablation of AF is increasingly used as a means to maintain sinus rhythm, and mounting evidence supports ablation as an effective rhythm control strategy in many patients with AF.^4^, ^5^, ^6^, ^7^ Despite this data, patients with AF from lower socioeconomic backgrounds may have reduced access to these procedures.

Social deprivation, manifested through economic and social marginalization, is associated with adverse outcomes in cardiovascular diseases. Evidence largely collected from countries, such as the United States, that have nonuniversal health care systems has shown that lower-income individuals and Black and Latino populations are less likely to receive access to ablation procedures.^8^, ^9^, ^10^, ^11^ However, given the nonuniversal nature of these health care systems, it is challenging to discern which dimensions of social deprivation are fueling issues related to access to catheter ablation, as ability to pay for care is a major barrier to access. Additionally, in prior studies related to ablation, access was not studied in the context of disease burden of AF, making it difficult to understand the relationship between these variables.

Accordingly, to address this gap in knowledge, we conducted a study in Canada, where health care is publicly funded and universal, allowing for the examination of social deprivation and its role in modulating access to care for patients with AF. This study aimed to address 2 research questions: first, what are the neighborhood rates of AF diagnosis (described in the study as disease burden), catheter ablation referrals, and catheter ablations, and what is the correlation between these variables? Second, what is the relationship between health inequities across 4 dimensions (dependency, material deprivation, ethnic concentration, and residential instability; using the 2016 version of the Ontario Marginalization Index) and rates of AF diagnosis, ablation referrals, and procedures? This study provides important insight into health care access issues for marginalized groups and is an important step in working toward policy interventions to ensure equitable AF care.

## Methods

### Context and cohort creation

This population-level retrospective cohort study was conducted in Ontario, Canada, a large and ethnically diverse province with a population of 15.6 million people, where health care is publicly funded and universally accessible.^12^ We adhered to the Strengthening the Reporting of Observational Studies in Epidemiology statement for the reporting of observational studies.^13^

This population-level study was conducted using administrative datasets linked to encoded identification codes and housed at the ICES (formally known as the Institute for Clinical Evaluative Sciences).^14^ Data utilization was authorized under Section 45 of Ontario’s Personal Health Information Protection Act, which does not require research ethics approval. Procedural data pertaining to catheter ablation was collected from the CorHealth Ontario Clinical Registry, a nonprofit agency that collects and stores all demographic, morbidity, and procedural data on invasive cardiac procedures in the province. The Canadian Institute for Health Information Discharge Abstract Database was used to collect data to supplement baseline characteristics and information related to comorbidities. Physician billing claims, as recorded in the Ontario Health Insurance Plan database, were used to identify patients with AF in the outpatient setting. Statistics Canada, a federal agency tasked with compiling census data, divides the province into 49 census divisions, which have distinct geographic boundaries. Indices for social deprivation are reported for each census division.

### Population

Using the linked datasets, all Ontario residents 18 years or older from April 1, 2016, to March 31, 2020, diagnosed with AF for the first time were included in the study. For inpatient records, AF diagnosis was defined as hospitalization with International Classification of Diseases 10th edition code I48 as the primary or secondary reason for hospitalization or a visit to the emergency department with the same code. To identify patients who are primarily treated in the outpatient setting, Ontario Health Insurance Plan billing data was used, and AF diagnosis was defined as 4 billing claims with code 427 in a period of 365 days. This combined definition of AF has been validated in previous studies and has a specificity of 99.1% for AF.^15^ The procedural cohort included patients 18 years of age or older and excluded patients who had an ablation procedure in the 5 years preceding the index date. For patients with multiple ablations during the study period, only the first recorded procedure was used.

### Outcomes

The outcomes of interest were: 1) disease burden; 2) ablation referral rate; and 3) ablation procedure rate within each census division. All rates were reported as incidence or procedures per 1,000 individuals per census division.

### Social deprivation

The primary exposures of interest were the 4 dimensions of social deprivation in the Ontario Marginalization Index (ON-MARG), stratified by quintile.^16^^,^^17^ The ON-MARG is a geography-based index that is derived from census data and can be used to measure health inequities in Ontario across 4 dimensions: dependency, material deprivation, ethnic concentration, and residential instability. The specific indicators used to qualify these dimensions can be found in the supplemental section (Supplemental Table 1). The dimensions are stratified by quintile, with higher quintile ranks corresponding to higher levels of marginalization. Each dimension is reported individually, and there is no composite score for the index. Given the study period, the 2016 version of the ON-MARG index was used, and the geographic unit of analysis used was the census division level (n = 49).^18^

### Statistical analysis

As this is a population-level study, descriptive statistics were used to present the data. To elucidate whether there was geographic variation across the province, heatmaps were constructed at the census division level for each outcome. Pearson correlation coefficients were calculated to further understand the association between disease burden and ablation referral and procedure rates. Four linear models with log link and Poisson distribution were constructed to both understand the relationship and the strength of the relationship between the outcomes of interest and each individual dimension of marginalization in the ON-MARG index. For each outcome of interest, the associations with the dimensions of the ON-MARG index were examined using separate univariable models, as suspected collinearity between the dimensions precluded the construction of a multivariable model. Individual-level characteristics such as age and sex were excluded as the unit of analysis was at a geographic level. All statistical analyses were performed using SAS Enterprise Guide 7.1 (SAS Institute Inc).

## Results

### Baseline characteristics

**Figure 1:**
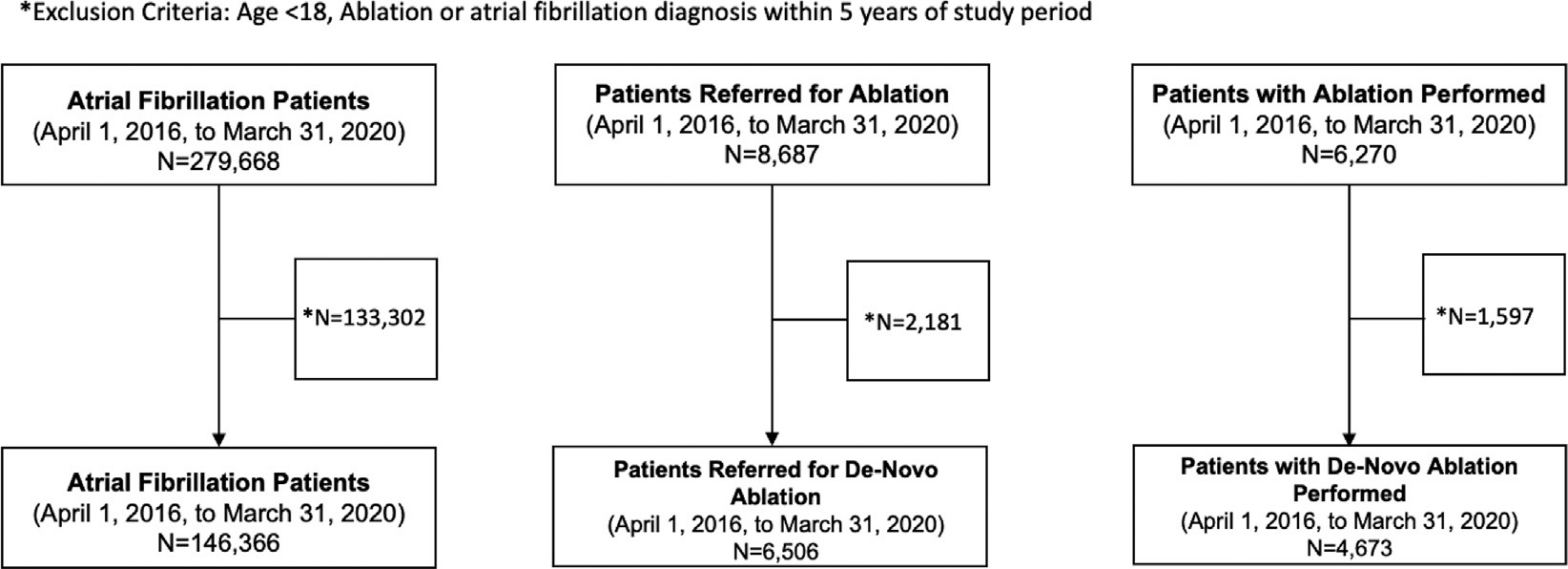
Atrial Fibrillation, Catheter Ablation Referral, and Procedural Cohorts Flowcharts depicting the number of patients included in each study cohort.

From April 1, 2016, to March 31, 2020, we identified 141,366 unique patients diagnosed with AF for the first time who met the inclusion criteria (Figure 1). Over the same time period, 6,506 patients were referred for a first-time or de novo AF ablation, and 4,673 patients underwent a first-time or de novo ablation procedure. The baseline characteristics of the patients are presented in Table 1. The median age of those who were referred for and received ablation was 62 years, which was lower than the median age of those diagnosed with AF, which was 72 years. Patients diagnosed with AF were more often males (54.7%), with a greater percentage of males referred for ablation (65.8%) and undergoing the procedure itself (66.8%). Patients with AF had on average higher Charlson comorbidity scores (0.59) than those referred for ablation (0.40) and those who received ablation (0.36).

**Table 1:** Baseline Characteristics of Study Cohorts

	Disease Burden (n = 141,366)	Ablation Referrals (n = 6,506)	Ablation Procedures (n = 4,673)
Age, y	72 (61-81)	62 (55-69)	62 (55-69)
Female	64,104 (45.3%)	2,227 (34.2%)	1,553 (33.2%)
Charlson comorbidity score	0.59	0.40	0.36
Frailty score	1.36	0.67	0.60
Frailty risk category			
High risk (≥5)	12,639 (8.9%)	207 (3.2%)	117 (2.5%)
Low risk (<5)	128,727 (91.1%)	6,299 (96.8%)	4,556 (97.5%)
Hypertension	99,116 (70.1%)	3,733 (57.4%)	2,686 (57.5%)
Diabetes	41,871 (29.6%)	1,100 (16.9%)	751 (16.1%)
Ischemic heart disease	12,851 (9.1%)	661 (10.2%)	473 (10.1%)
Vascular disease	20,362 (14.4%)	762 (11.7%)	518 (11.1%)
Congestive heart failure	28,527 (20.2%)	1,228 (18.9%)	832 (17.8%)
Prior MI	4,191 (3.0%)	155 (2.4%)	98 (2.1%)
Prior stroke	2,260 (1.6%)	80 (1.2%)	59 (1.3%)
Cerebrovascular disease	3,458 (2.4%)	113 (1.7%)	78 (1.7%)
Peripheral vascular disease	1,374 (1.0%)	26 (0.4%)	13 (0.3%)
Dyslipidemia	54,247 (38.4%)	2,059 (31.6%)	1,497 (32.0%)
Chronic kidney disease	3,055 (2.2%)	61 (0.9%)	32 (0.7%)
Dialysis	2,088 (1.5%)	10 (0.2%)	7 (0.1%)
Cancer	7,097 (5.0%)	147 (2.3%)	91 (1.9%)
Liver disease	1,193 (0.8%)	31 (0.5%)	24 (0.5%)
Interstitial lung disease	928 (0.7%)	23 (0.4%)	11 (0.2%)
Dementia	10,070 (7.1%)	35 (0.5%)	23 (0.5%)
Previous CABG	8,006 (5.7%)	170 (2.6%)	96 (2.1%)
Previous PCI	12,145 (8.6%)	443 (6.8%)	319 (6.8%)
Previous valve surgery	4,368 (3.1%)	137 (2.1%)	72 (1.5%)
Previous pacemaker	4,738 (3.4%)	197 (3.0%)	150 (3.2%)
Previous ICD	2,153 (1.5%)	127 (2.0%)	81 (1.7%)

Values are median (IQR) or n (%).

CABG = coronary artery bypass graft; ICD = implantable cardioverter defibrillator; MI = myocardial infarction; PCI = percutaneous coronary intervention.

### Regional variation

**Figure 2:**
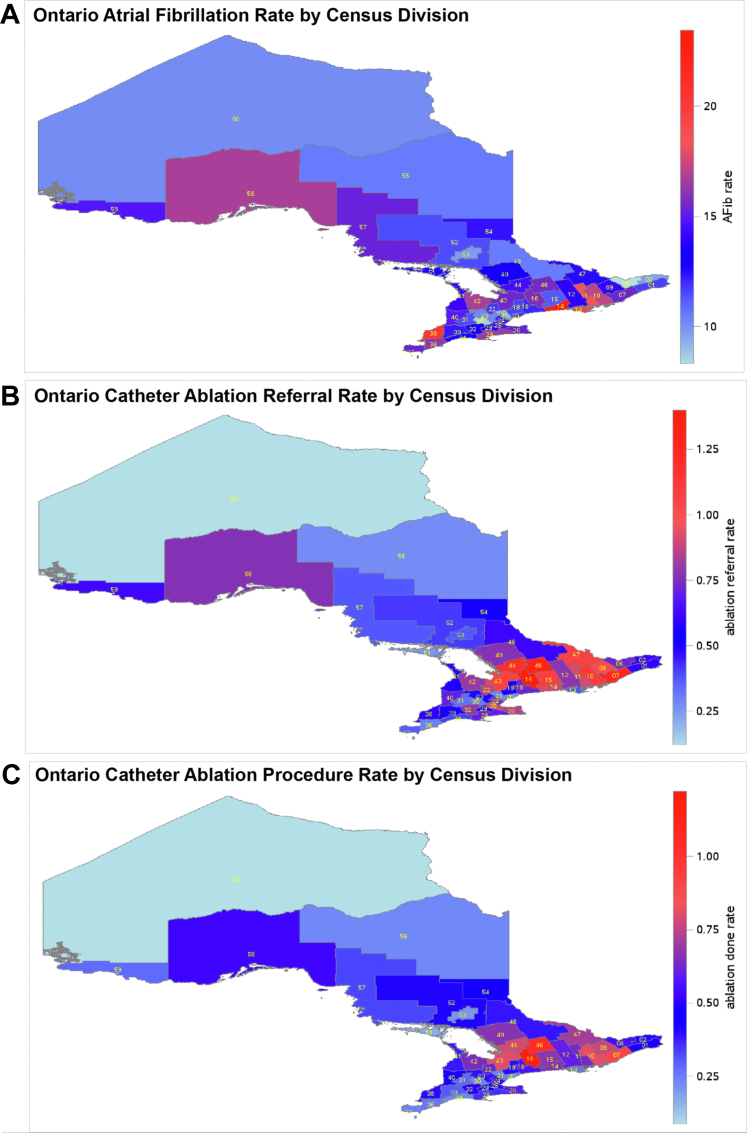
Heat Maps Incidence of study cohorts per 1,000 individuals by census division. (A) Atrial fibrillation diagnosis; (B) catheter ablation referrals; (C) catheter ablation procedures.

**Figure 3:**
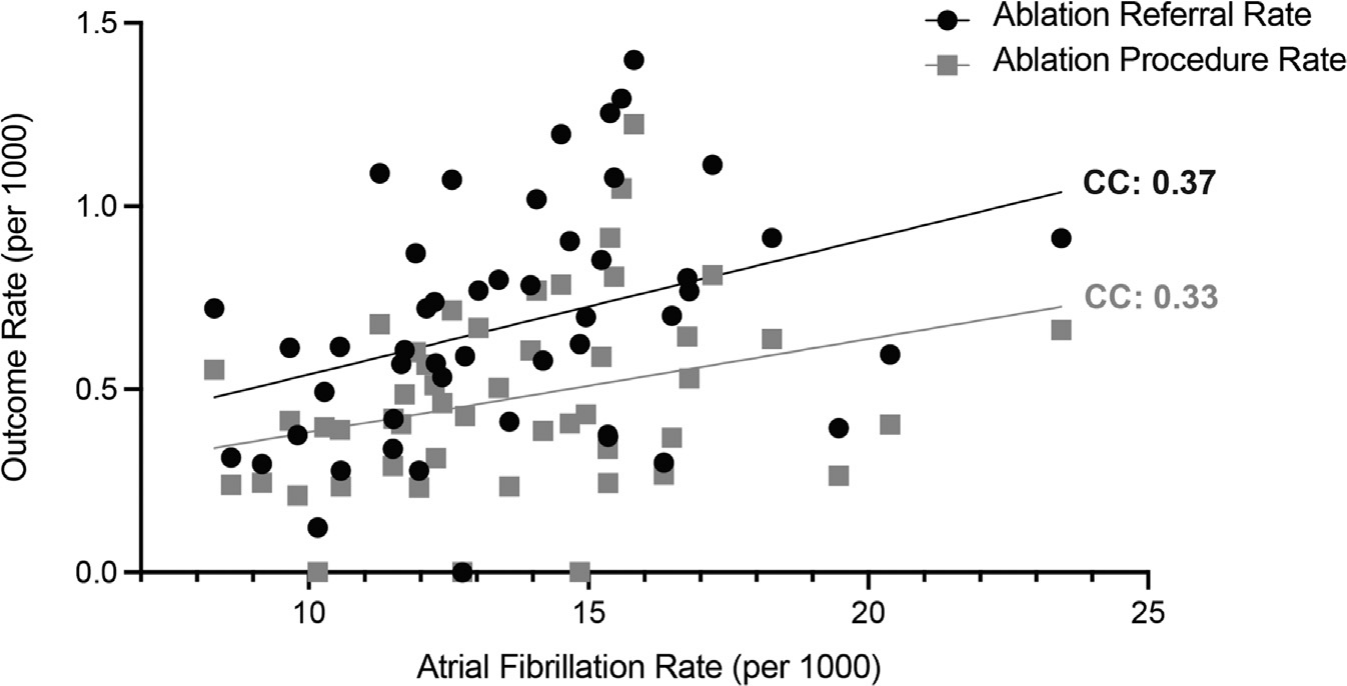
Correlation Scatter Plot Correlation between disease burden of atrial fibrillation and catheter ablation referral and procedure rate, using Pearson correlation coefficients.

There was wide geographic variation of disease burden, ablation referral, and procedural rates across census divisions (Figure 2). Geographic variations between disease burden and ablation referrals and procedures were weakly concordant (Pearson correlation coefficient 0.37 and 0.33, respectively; Figure 3). In contrast, the correlation between ablation referral rates and procedure rates was 0.95.

### Social deprivation

**Figure 4:**
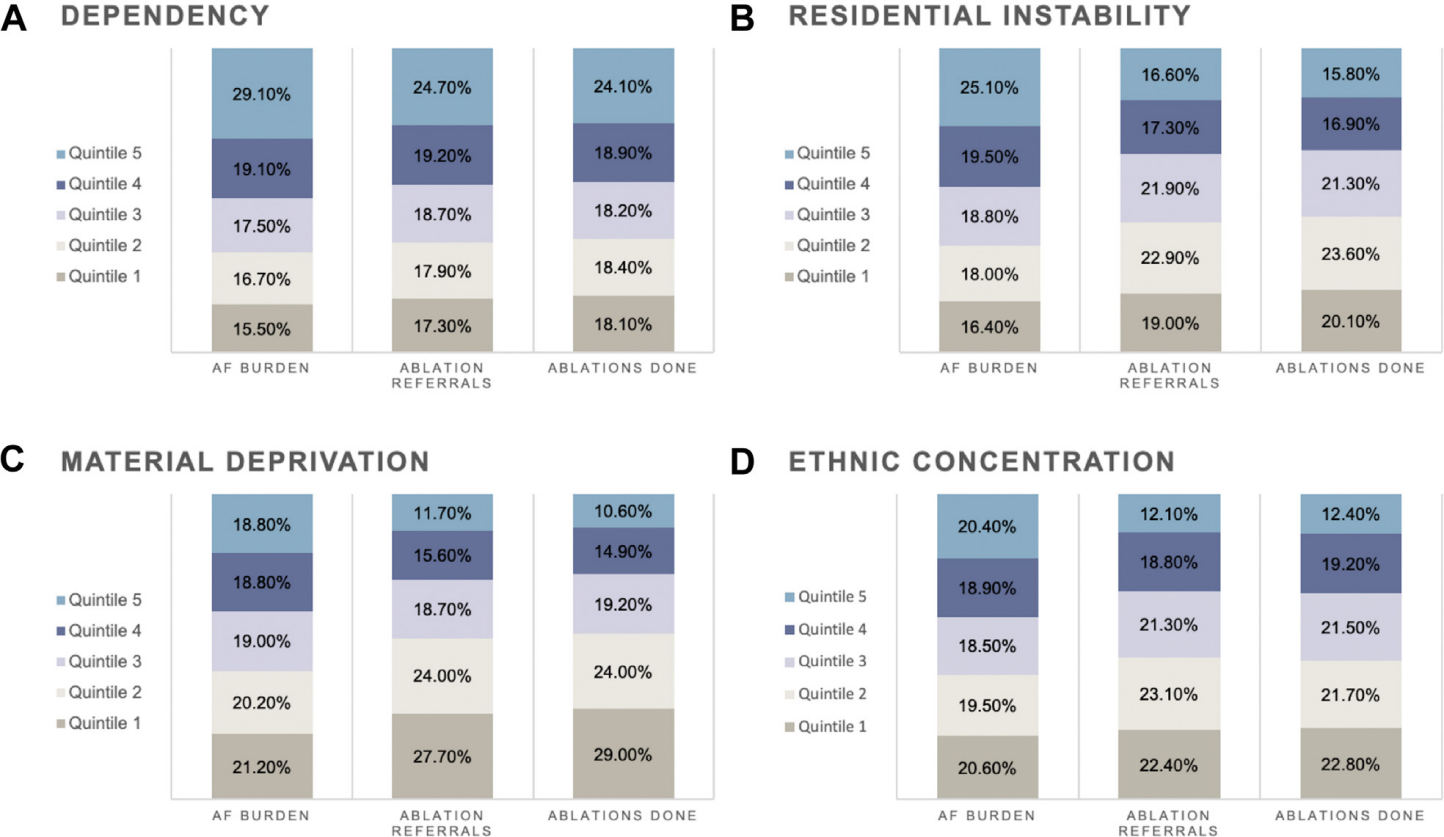
Census Divisions in Each Quintile of Social Deprivation Dimensions Census division distribution across dimensions of social deprivation (A) dependency (B) residential instability (C) material deprivation (D) ethnic concentration and outcomes of interest.

The proportion of census divisions in each quintile of the ON-MARG dimensions is depicted in Figure 4 and is further stratified by the outcomes of interest. There was marked variation in the proportions of census divisions making up the quintiles of the ON-MARG dimensions.

For ethnic concentration, the highest quintile of marginalization made up 20.4% of disease burden, but only 12.1% of ablation referrals and 12.4% of ablations performed. Similarly, material deprivation at the highest quintile of marginalization accounted for 18.8% of disease burden, but only 11.7% and 10.6% of ablation referrals and ablations performed, respectively. Residential instability and dependency demonstrated similar trends.

**Figure 5:**
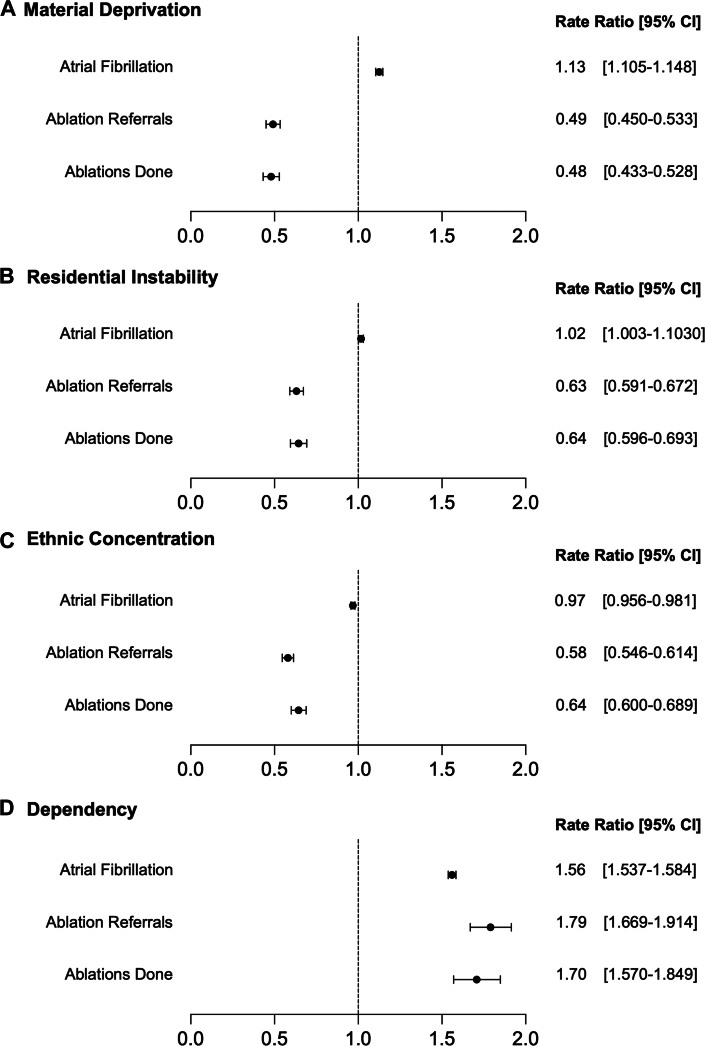
Regression Models Univariable models of association between (A) material deprivation (B) residential instability (C) ethnic concentration (D) dependency and atrial fibrillation disease burden, ablation referrals, and ablation procedures.

Figure 5 depicts the regression models showing the relationship between the dimensions of social deprivation and disease burden and access. As seen in Figure 5A, increasing material deprivation was associated with higher rates of disease burden (rate ratio [RR]: 1.13; 95% CI: 1.11 to 1.15), but lower rates of ablation referrals (RR: 0.49; 95% CI: 0.45-0.53) and ablations performed (RR: 0.48; 95% CI: 0.43-0.53). Similarly, residential instability had a discordant relationship as seen in Figure 5B; it was associated with slightly increased rates of disease burden (RR: 1.02; 95% CI: 1.003-1.030), but lower rates of both ablation referrals (RR: 0.63; 95% CI: 0.59-0.67) and procedures (RR: 0.64; 95% CI: 0.60-0.69). Ethnic diversity (Figure 5C) was associated with lower rates of disease burden, ablation referrals, and ablations performed, while dependency (Figure 5D) was associated with higher rates of disease burden, ablation referrals, and ablations performed.

Interestingly, when looking at patients of the female sex, increased ethnic diversity was associated with higher rates of AF (RR: 1.07; 95% CI: 1.06-1.08) and higher rates of ablation referrals (RR: 1.28; 95% CI: 1.22-1.34) and ablations performed (RR: 1.25; 95% CI: 1.18-1.32). This model is depicted in the supplemental section (Supplemental Figure 1).

## Discussion

This population-level cohort study assessed the impact of marginalization on disease burden of AF and access to catheter ablation in a publicly funded health care system with universal access to care. There was wide geographic variation in disease burden and ablation referral and procedure rates across the province. Indices of marginalization and social deprivation, including material deprivation, ethnic concentration, dependency, and residential instability, all played an important role in modulating access to AF care, albeit contrasting ones (Central Illustration).

**Central Illustration:**
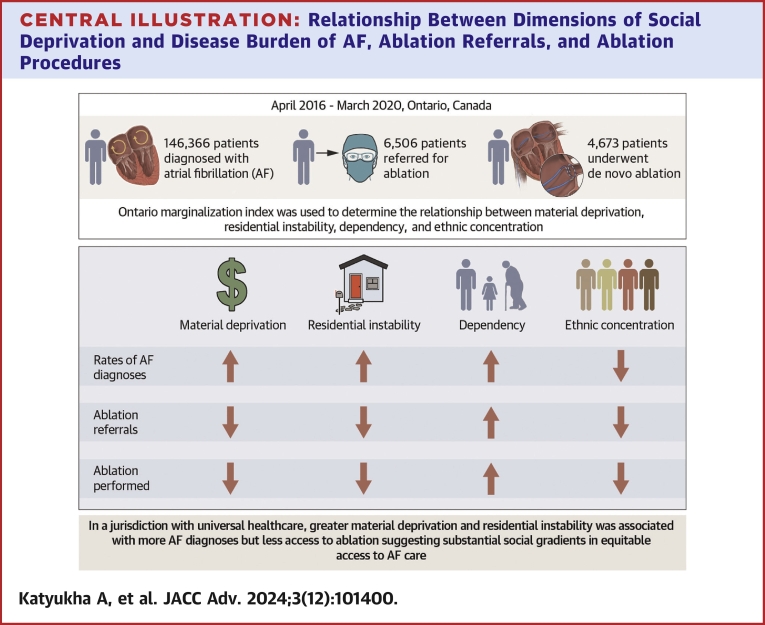
Relationship Between Dimensions of Social Deprivation and Disease Burden of AF, Ablation Referrals, and Ablation Procedures

Several studies have reported on the geographic variation and variable access to ablations in Canada and the United States,^11^^,^^19^^,^^20^ but the reason behind variable access remains unclear. We reported a relatively low concordance between disease burden and both ablation referrals and ablations performed, suggesting there are important factors that impact the decision to refer patients for catheter ablation aside from burden of disease. Indeed, the dimensions of social deprivation may have a substantial impact on ablation referrals and ablations performed.

When we analyzed data across the dimensions of social deprivation, a number of trends emerged that can help inform areas of focus for intervention to improve access to ablations.

### Dependency

Increased dependency, which is largely representative of increased age, a risk factor for AF,^21^ was associated with an increased incidence of AF diagnosis and concordantly higher rates of ablation referrals and ablations performed.

### Material deprivation

Increased material deprivation, which reflects poverty and inability to access basic resources such as food, housing, and education, was associated with higher rates of AF diagnosis but lower rates of ablation referrals and ablations performed and are consistent with data from smaller studies.^19^ Lower socioeconomic status has been reportedly associated with lower rates of ablation referrals,^9^^,^^11^ despite patients having significant symptom burden.^22^^,^^23^ Furthermore, lower education levels are associated with lower health literacy,^24^ potentially limiting patients’ knowledge and understanding of more complex treatments such as ablation. While prior studies have focused on nonuniversal health care systems, our findings demonstrate that accessibility issues persist even in a publicly funded universal health system.

### Residential instability

Increased residential instability, which reflects reduced family and neighborhood stability and cohesiveness, was associated with higher rates of disease burden but lower ablation referral and ablations performed. Social isolation may impact patients’ mental health,^25^ thereby limiting their interaction with the health care system and ability to adequately follow-up after the initial AF diagnosis. Moreover, receiving expert care in centers that offer ablation procedures may require longer time commitments, deferring care to family members, and those with greater residential instability may experience reluctance for procedural interventions due to a lack of emotional and caregiver support.^26^^,^^27^

### Ethnic concentration

Increased ethnic concentration, which measures the proportion of newcomers and non-White, non-Indigenous populations in a certain region, was associated with lower rates of disease burden, ablation referral, and procedure rates. This is a similar finding that was demonstrated in a study examining aortic stenosis and surgical and transcatheter aortic valve replacement rates in our jurisdiction.^28^ Our study was not structured to determine the underlying reason for this finding, but as this dimension includes immigrant populations the “healthy immigrant” effect may mediate some of these findings. Individuals immigrating to Canada may be younger and healthier than the general population and, as a result, have lower rates of AF to begin with, thereby also reducing the number of ablation referrals.^29^ An alternate explanation is that these populations may not readily navigate the healthcare system in Ontario,^30^ leading to fewer AF diagnoses and ablation referrals. This idea is supported by the fact that Ontario has reported a primary care physician shortage for the duration of this study.^31^

Interestingly, when looking at data pertaining to females in areas of increased ethnic concentration, females had higher rates of disease burden, ablation referral, and procedure rates, contrasting the results described above. Though traditionally males were thought to be at an increased risk for developing AF, when compared to females, new research has shown that females have up to a 50% higher rate of AF diagnosis, when height is controlled among participants.^32^ Moreover, previous studies have shown that females tend to have more symptom burden,^33^^,^^34^ although prior research has shown that females are overall less likely to be referred for invasive procedures.^35^^,^^36^ The contrasting relationship between sexes and ethnicity requires further study.

This study adds to our understanding of social deprivation in AF care and reinforces the need to consider how socioeconomic status and marginalization impact our patients’ access to cardiac procedures. Even though our jurisdiction offers publicly funded, universally accessible health care, there are structural barriers that are tangibly affecting the care patients receive, and efforts must be made to bridge the gap for marginalized patients. Our findings also underscore the fact that these dimensions of social deprivation can lead to biases that substantially impact ablation referral patterns,^37^^,^^38^ and thus, public health interventions are required to identify and ameliorate these biases.

### Future directions

Building on this study, we can determine which parts of the province experience the most marginalization and evaluate the feasibility of expanding care in those communities. This can be done by exploring interventions such as satellite care networks that allow individuals in those communities to access specialized care, and referral guidelines for primary care providers practicing in affected communities, where specialist care may not be readily available. This study aims to highlight the broader trends affecting access to care for marginalized patients in the hopes of encouraging further research aimed at identifying solutions to these important issues.

### Study Limitations

Given the geographic unit of our data (census division), we were not able to include patient-specific characteristics in our analysis. Though this is mitigated by the fact that certain dimensions, such as age and income, are incorporated into the dimensions of material deprivation and dependency, we do not know the impact of patient-specific or disease-specific (ie, AF subtype) characteristics on our trends. Furthermore, due to suspected collinearity between variables in the ON-MARG index, we could not create a multivariable model to account for various indices of social deprivation or their interactions. Additionally, as this is a population-level study, these results are only meant to describe the population studied. Our findings are at risk of ecologic fallacy, and caution needs to be taken to not draw conclusions about individuals based on the groups they belong to.^39^

## Conclusions

In a large, ethnically diverse jurisdiction with publicly funded, universally accessible health care, patients living in regions with high levels of residential instability and material deprivation have an increased disease burden of AF but are less likely to access catheter ablation. Data from this study can be used to further examine specific barriers in accessing ablation care for patients with AF and to create public health interventions targeted at bridging the gap for marginalized communities in the hopes of building a more just and equitable health care system.

Perspectives **COMPETENCY IN SYSTEMS-BASED CARE:** Despite evidence that catheter ablation is an effective strategy for rhythm control in AF, patients living in areas experiencing social deprivation are less likely to access these therapies. Further work needs to be taken to ensure health systems are providing equitable and high-quality care for patients, and as physicians, we need to continue quality improvement initiatives and research that impacts policies that increase access to care.

**TRANSLATIONAL OUTLOOK:** A number of barriers exist in ensuring equitable access to catheter ablation for AF, and research aimed at addressing policy changes to improve access will ensure better clinical outcomes for patients. Continued emphasis on gathering data related to sex, gender, and factors related to social deprivation will allow for improvements in health delivery and outcomes.

## Funding support and author disclosures

This study was supported by ICES, which is funded by an annual grant from the Ontario 10.13039/100009647Ministry of Health (MOH) and the Ministry of Long-Term Care (MLTC). Dr Wijeysundera is supported by a 10.13039/501100001804Canada Research Chair in Structural Heart Disease Policy and Outcomes. The authors have reported that they have no relationships relevant to the contents of this paper to disclose. This document used data adapted from the Statistics Canada Postal Code OM Conversion File, which is based on data licensed from Canada Post Corporation, and/or data adapted from the Ontario Ministry of Health Postal Code Conversion File, which contains data copied under license from Canada Post Corporation and Statistics Canada. Parts of this material are based on data and information compiled and provided by the Canadian Institute of Health Information (CIHI) CorHealth Ontario and the Ministry of Health. The analyses, conclusions, opinions, and statements expressed herein are solely those of the authors and do not reflect those of the funding or data sources and CIHI; no endorsement is intended or should be inferred.
