# Supplementary material for: Relationship Between Social Deprivation and Access to Catheter Ablation for Atrial Fibrillation: A Population-Level Study
Source: JACC Adv. 2024 Nov 18;3(12):101400. doi: 10.1016/j.jacadv.2024.101400 (PMC11612362; doi:10.1016/j.jacadv.2024.101400)

Supplemental Table 1. Indicators making up the Ontario Marginalization Index dimensions.

| **Residential Instability** |
| --- |
| Proportion of the population living alone |
| Average number of persons per dwelling |
| Proportion of dwellings that are apartment buildings |
| Proportion of population who are single/divorced/widowed |
| Proportion of dwellings that are not owned |
| Proportion of population who moved during past 5 years |
|  |
| **Material Deprivation** |
| Proportion of population aged 20+ without a high school diploma |
| Proportion of families who are lone parent families |
| Proportion of total income from government transfer payments for population aged 15+ |
| Proportion of population aged 15+ who are unemployed |
| Proportion of the population considered low-income |
| Proportion of households living in dwellings in need of major repair |

| **Dependency** |
| --- |
| Proportion of population who are aged 65 and older |
| Dependency ratio (total population 0-14 and 65+/total population 15 to 64) |
| Proportion of population not participating in labour force (age 15+) |

| **Ethnic Concentration** |
| --- |
| Proportion of population who are recent immigrants (arrived in the past 5 years) |
| Proportion of the population who self-identify as a visible minority |

Supplemental Figure 1. Univariable model of association of between ethnicity and disease burden of AF catheter ablation referral and procedural rates, controlled for female sex.


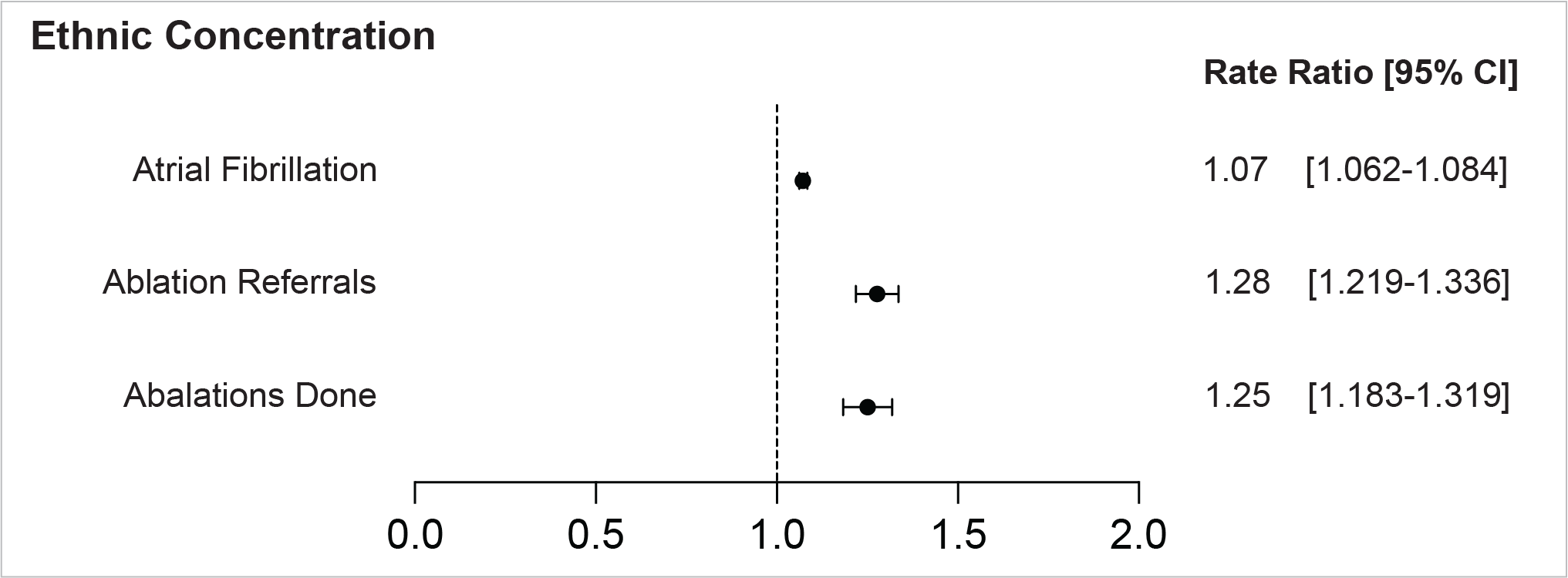

Supplement: Supplemental material [file mmc1.docx]
